# The advantages of model fitting compared to model simulation in research on preference construction

**DOI:** 10.3389/fpsyg.2015.00140

**Published:** 2015-02-18

**Authors:** Edgar Erdfelder, Marta Castela, Martha Michalkiewicz, Daniel W. Heck

**Affiliations:** Cognition and Individual Differences Lab, Department of Psychology, University of MannheimMannheim, Germany

**Keywords:** multinomial modeling, model fitting, model simulation, preference construction, serial-position effects, primacy and recency effects

Mantonakis et al. (2009) suggested the pairwise-competition model (PCM) of preference construction. The basic idea is that humans form preferences among choice objects (e.g., types of wines) sequentially, such that the preferred object is the ultimate winner of a sequence of pairwise competitions: The object sampled first is compared to the second, the winner of this competition is then compared to the third object, resulting in a winner that is compared to the fourth, and so on. For a set of *m* objects, this recursive process terminates after *m*-1 pairwise competitions once the final object has been compared against the previous favorite. Canic and Pachur (2014) recently proposed a formal specification and elaboration of the PCM. Moreover, using computer simulations, they showed how variations in the model's parameters affect primacy and recency effects in sequential choice and how the model can account for various patterns of serial position effects documented in the literature.

We appreciate the contributions of Mantonakis and collaborators as well as Canic and Pachur. Clearly, innovative theoretical ideas and their formal clarification are key elements of progress in science. However, we argue that making use of the full spectrum of formal modeling advantages necessarily involves *fitting the model to empirical data*. By narrowing down model-based analyses to *computer simulations*—as Canic and Pachur (2014) did—important questions remain unanswered and misleading conclusions cannot be ruled out.

First, simulating a model and showing that it predicts serial-position effects similar to those found in empirical data does not imply that the model fits these data. A successful model should account for the entire distribution of observed data, not just for selected qualitative aspects such as primacy and recency effects. Formal goodness-of-fit tests provide the necessary information but simulations do not.

Second, if there are several nested versions of a model as in case of the PCM, simulations cannot tell us which model version provides the best account of the data taking model complexity into account (as influenced, among others, by the number of estimated parameters). The model fitting toolbox, in contrast, includes appropriate model selection measures, enabling us to identify the most parsimonious model version that is consistent with the data.

Third, model fitting involves parameter estimation, that is, identification of the parameter values that best account for an existing set of data. It also provides statistical tests for parameters, enabling statistically sound evaluations of parameter differences between groups or conditions. Simulations, in contrast, do not provide such an elaborated framework. Because they ignore sampling variability in empirical data, they may mistakenly suggest parameter differences when in fact there are none.

To illustrate the advantages of model fitting, we apply Canic and Pachur's (2014) formalization of the PCM to the data of Mantonakis and collaborators^1^.

## The PCM as a multinomial processing tree model

Fortunately, fitting the PCM to empirical data is straightforward. Inspection of the model equations reveals that the PCM belongs to the class of multinomial processing tree (MPT) models (Batchelder and Riefer, 1999), a model family with a well-developed statistical theory (Hu and Batchelder, 1994) that has already been implemented in easy-to-use software tools (Moshagen, 2010; Singmann and Kellen, 2013). MPT models are models for categorical data, such as frequencies *f_m,j_* of preferring the *j*-s object in the sequence, *j* = 1, …, *m*, given the total number of objects is *m*. They assume a joint multinomial distribution of the frequencies with underlying category probabilities that correspond to sums of products of *S* model parameters θ_s_ and their complements (1-θ_s_), *s* = 1, …, *S*, each being an element of the unit interval [0, 1]. The basic MPT model architecture has been found useful to represent sequences of discrete cognitive processes underlying task performance (for a review, see Erdfelder et al., 2009) and, more generally, relations between latent psychological states and categorical test results (e.g., Botella et al., 2013; Erdfelder and Moshagen, 2013).

To formalize the PCM within the MPT framework, we first consider the simplest special case of choosing among *m* = 2 objects. According to the PCM, humans show “choice inertia” and stick to their previous favorite (i.e., the first object) with probability π, thus avoiding any comparison. With the complementary probability (1-π), they enter the competition state, in which the second object wins with probability *p*(new) (determined by Luce's choice rule) and, by implication, the first one with probability 1-*p*(new). Thus, preference for the first object can result from choice inertia (probability π) or from the first object winning against the second when choice inertia is absent (probability (1-π)(1-*p*(new))). The overall probability of choosing the first object is the sum of these probabilities:

(1) $$\begin{array}{l} p(\text{object }1)=\\ \;\pi +\left(1-\pi \right)\cdot \left(1-\;p(\text{new})\right)\text{​}. \end{array}$$

In contrast, preference for the second object can result from a single sequence only:

(2) p(object 2)=(1−π)·p(new).

These equations match the basic MPT structure. Generalization to choice among three or more objects is straightforward because this only involves recursive applications of the processes already described (although not necessarily with the same probabilities). The corresponding model specification file required for analyzing the data of Mantonakis and collaborators using the multiTree program (Moshagen, 2010) can be requested from the first author. It provides for a 2-factorial design (factor A: Number of wine samples in the choice set: 2–5; factor B: Knowledge about wines: high vs. low) as implemented by Mantonakis et al. (2009).

## Fitting the PCM to the data of Mantonakis et al. (2009)

Mantonakis et al. (2009) assigned their 142 participants (69 people with high and 73 people with low knowledge about wines) randomly to 4 groups receiving choice sets consisting of 2–5 wine samples, respectively. Following the tasting sequence, each participant indicated her or his preferred wine in the set. Unbeknownst to the participants, the same wine was actually used in each sample. Thus, it seems reasonable to constrain the probabilities *p*(new)*_j_*, *j* = 2, …, 5, that the *j*-s wine sample wins the competition against the current favorite to *p*(new)*_j_* = 0.5 for all participants and choice sets *j*. Our first version of the model, PCM(1), is based on this assumption only. Consequently, all choice inertia parameters π_j_, *j* = 2, …, 5, are left unconstrained for both high and low-knowledge participants. This model accounts for 2 · (1 + 2 + 3 + 4) = 20 independent choice probabilities across the 2 · 4 = 8 groups by 8 choice inertia parameters and is identifiable. It fits the observed choice frequencies for wine experts, *G*^2^(6) = 5.63, *p* = 0.47, non-experts, *G*^2^(6) = 3.97, *p* = 0.68, and for both samples analyzed conjointly, *G*^2^(12) = 9.60, *p* = 0.65.

**Figure 1:**
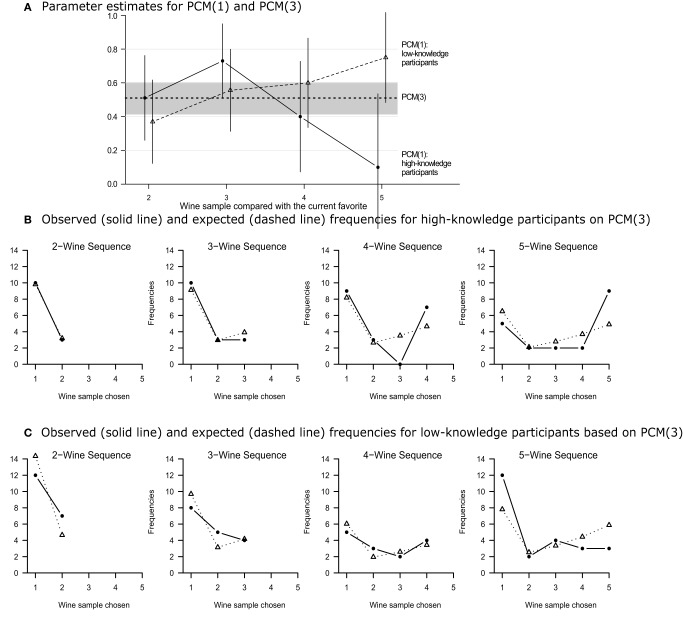
**(A) Estimates of the choice inertia parameters of models PCM(1) and PCM(3)**. Vertical lines indicate 95% confidence intervals for the eight parameters of PCM(1). The gray area illustrates the 95% confidence interval of the single PCM(3) choice inertia parameter. **(B,C)** Observed (solid lines) and expected frequencies (dashed lines) for the 1-parameter PCM(3) model applied to the choice frequencies of Mantonakis et al.'s (2009) high vs. low knowledge participants, respectively.

Figure 1A displays the maximum likelihood (ML) estimates of the π *_j_* along with the 95% confidence intervals. Descriptively, there appears to be an inverted u-shaped trend for experts and a monotonically increasing trend for non-experts with increasing sequence lengths. However, an equality constraint on the 4 inertia parameters (i.e., π_2_ = π_3_ = π_4_ = π_5_) does not decrease the fit significantly, neither for experts, Δ*G*^2^(3) = 7.07, *p* = 0.07, nor for non-experts, Δ*G*^2^(3) = 3.93, *p* = 0.27, nor for both groups analyzed conjointly, Δ*G*^2^(6) = 11.00, *p* = 0.09. The second model version, PCM(2), includes both constraints and shows an excellent overall fit, *G*^2^(18) = 20.61, *p* = 0.30. Surprisingly, the choice inertia estimate is only slightly lower for high-knowledge compared to low-knowledge participants (0.485 vs. 0.532, respectively), a difference that is far from being significant, Δ*G*^2^(1) = 0.21, *p* = 0.64. This suggests an even more restrictive model version PCM(3) with all eight inertia parameters equated. With a single remaining choice inertia estimate for both groups (0.510, shown as a constant line in Figure 1A), PCM(3) provides an excellent overall fit, *G*^2^(19) = 20.82, *p* = 0.35.

Given that all three models fit the data nicely it must be expected that the model with the fewest free parameters performs best in terms of model selection criteria that penalize model complexity. Because standard methods such as the Akaike and the Bayesian information criterion (AIC and BIC) focus on the number of free parameters only and ignore differences in the functional form of the models, we used the more sophisticated Fisher information approximation (FIA; Wu et al., 2010). FIA can safely be applied in the present case because the Mantonakis sample exceeds Heck's lower bound for FIA applications (i.e., *N* = 142 > *N*′ = 16; see Heck et al., 2014). The FIA values for the models PCM(1), PCM(2), and PCM(3) were 166.4, 165.4, and 163.8, respectively, showing that PCM(3) performs best. In order to better judge the evidence for each of the three models, we computed the relative weights *w*_FIA_ (i.e., estimates of the probabilities of each model being the best one in the set). The FIA weights 0.056, 0.158, and 0.786 for the models PCM(1), PCM(2), and PCM(3) highlight that a single inertia parameter suffices to account for the data.

In sum, both goodness-of-fit tests and model selection criteria suggest that PCM(3) is the best model, despite the fact that this model assumes neither serial position effects for π *_j_* and *p*(new)*_j_* nor differences in parameters between high- vs. low-knowledge participants. As illustrated in Figures 1B,C, this model accounts for the primacy and recency effects in the preference curves of high and low knowledge participants, respectively, although it includes a single inertia parameter only.

## Discussion

What do we learn from this model fitting exercise? First, we can now say that the PCM indeed fits the entire distribution of preferential choice data obtained by Mantonakis et al. (2009), not just the primacy and recency effects discussed by Canic and Pachur (2014). Somewhat surprisingly, however, the model requires much less parameters to account for order effects in preferential choice data than hypothesized by Mantonakis et al. (2009) and proposed by Canic and Pachur (2014) based on their simulation results. As shown here, there is no evidence for significant differences in choice inertia, neither across the course of the choice sequence nor between high- and low-knowledge participants. Rather, both groups of participants exhibit choice inertia roughly 50% of the time. Of course, we cannot rule out that this picture would change for groups with larger knowledge differences than in the Mantonakis sample. Moreover, the moderate sample size may play a role: Given a significance level of α = 0.05 and the current *N* = 142, the power of the *G*^2^(1) goodness-of-fit test is appropriate for medium deviations from the null hypothesis (i.e., *w* = 0.3; 1-β = 0.95) but not for small deviations (i.e., *w* = 0.1; 1-β = 0.22). A sample more than five times larger than the Mantonakis sample (specifically, *N* = 785) would be necessary to achieve a power of 0.80 in the latter case (Faul et al., 2009). Given the present data, however, nothing more can be said than that a single free parameter suffices to fit them well.

Future research should conduct stronger tests of the PCM than presently possible, both by using larger samples and by applying the model not just to the final preferences but to the entire vector of (preliminary) preferences during the tasting sequence. Unfortunately, although it is straightforward to generalize the PCM accordingly, appropriate process data for evaluating such a generalized PCM are currently unavailable. In addition, it would be desirable to generalize the PCM to a multilevel model that captures individual variability in parameters. Again, statistical frameworks for such a generalization are available (e.g., Klauer, 2006, 2010) but appropriate data (including several preference judgments per participant) are still lacking. Last but not least, one might think about comparing the PCM to alternative models of order effects in preference data, for example, models based on signal-detection theory for forced-choice judgments (e.g., DeCarlo, 2012).

To conclude, given the presently available data of Mantonakis et al. (2009), the up-to-date model fitting procedures that we relied on do not confirm conjectures about sequential trends or knowledge effects in choice inertia parameters as suggested by Canic and Pachur's simulation results. However, they do support the conclusion that Canic and Pachur's formal implementation of the PCM is an empirically successful model of preference formation.

### Conflict of interest statement

The reviewer David Kellen declares that, despite having collaborated with authors Marta Castela and Edgar Erdfelder, the review process was handled objectively and no conflict of interest exists. The authors declare that the research was conducted in the absence of any commercial or financial relationships that could be construed as a potential conflict of interest.

## Footnotes

^1^We are grateful to Antonia Mantonakis for providing the raw frequencies.
